# Hypoxia-inducible factor-1α and Wnt/β-catenin signaling pathways promote the invasion of hypoxic gastric cancer cells

**DOI:** 10.3892/mmr.2015.3812

**Published:** 2015-05-21

**Authors:** HONG-LAN LIU, DANG LIU, GUANG-RONG DING, PENG-FEI LIAO, JUN-WEN ZHANG

**Affiliations:** Department of Gastroenterology, The First Affiliated Hospital of Chongqing Medical University, Chongqing 400016, P.R. China

**Keywords:** hypoxia-inducible factor-1α, β-catenin, microRNA, urokinase-type plasminogen activator, matrix metalloproteinase-7

## Abstract

The present study aimed to examine the association between hypoxia-inducible factor (HIF)-1α and the Wnt/β-catenin signaling pathway in a hypoxic environment. The study also aimed to explore the possible mechanisms underlying the invasion of hypoxic gastric cancer cells in vitro and in vivo. The pcDNA™ 6.2-GW/EmGFP-miR-β-catenin plasmid was transfected into SGC-7901 gastric cancer cells, resulting in cells with stable suppression of β-catenin expression. The biological characteristics of the control, liposome, negative control, β-catenin knockdown, hypoxia and hypoxia β-catenin knockdown groups were tested using an invasion assay. The differences in the invasive capacity of the control, negative control and liposome groups were not statistically significant. However, the hypoxia group demonstrated a significantly enhanced invasive capacity, as compared with that in the control group (P<0.05). In the hypoxia β-catenin knockdown group, reduced cell penetration and diminished invasive behavior was observed (P<0.05). In the hypoxia and double (chemical + physical) hypoxia groups, HIF-1α, β-catenin, urokinase-type plasminogen activator (uPA) and matrix metalloproteinase (MMP-7) protein and mRNA expression levels were elevated. In response to knockdown of β-catenin expression, HIF-1α, β-catenin, uPA and MMP-7 protein as well as mRNA expression levels were significantly reduced in the hypoxia β-catenin knockdown and the double hypoxia β-catenin knockdown groups. In an in vivo experiment, the growth rate of xenograft tumors of hypoxic and control cells was high alongside increased HIF-1α, β-catenin, uPA and MMP-7 levels according to western blot and immunohistochemical analyses, while growth and protein levels of tumors from hypoxic β-catenin knockdown cells were significantly lower and those of β-catenin knockdown cells were lowest. In conclusion, these results suggested that HIF-1α activation was able to regulate the Wnt/β-catenin pathway, and that HIF-1α may be controlled by the Wnt/β-catenin pathway. A potential mechanism underlying SGC-7901 tumorigenicity is the activation of the Wnt/β-catenin signaling pathway, which activates uPA and MMP-7 expression and contributes to the enhanced invasion of hypoxic cancer cells.

## Introduction

The unlimited proliferation of tumor cells can cause hypoxia in tumor tissue. Hypoxia-inducible factor (HIF)-1α is an important transcription factor that regulates oxygen homeostasis, and may be associated with the occurrence of gastric cancer (1,2). HIF-1α is able to interact with specific signaling pathways that have important roles in adapting to hypoxia in tumor cells. In gastrointestinal tumors, the Wnt/β-catenin signaling pathway has been shown to affect cell proliferation, differentiation, and the regulation of microenvironment adaptability. HIF-1α competes with Tcf-4 for binding to β-catenin, and once bound it can activate HIF-1 target genes (3). Tumor cells generated in specific microenvironments have increased viability and can easily adapt to hypoxia (4) through activation of the canonical Wnt/β-catenin signaling pathway, which may lead to gastrointestinal tumorigenesis (5,6).

Invasion and metastasis involve numerous proteins from the matrix metalloproteinase (MMP) enzyme family (7,8). MMP-7 possesses strong matrix degradation activity, broad substrate specificity and has been shown to be overexpressed in invasive digestive cancers (9,10). In addition, the uroki-nase-type plasminogen activator (uPA) system can mediate cell-surface plasminogen activation, which can lead to degradation of the extracellular matrix and indirect activation of MMPs, which results in further degradation of extracellular matrix components (11). The expression of MMPs is associated with the micrometastasis of gastric cancer cells and poor prognosis, due to the important roles of these proteins in invasion and metastasis (12).

The present study examined the association between HIF-1α and Wnt/β-catenin in vitro in the SGC-7901 gastric cancer cell line. Furthermore, the signaling involved in metastasis and invasion was determined in vitro and in vivo under hypoxic conditions in SGC-7901 gastric cancer cells. The results of the present study may help to identify molecular targets for improved gastric cancer therapeutic strategies.

## Materials and methods

### Cell culture, plasmids and reagents

The SGC-7901 gastric cancer cell line was obtained from the Chongqing Medical University Key Laboratory of Neurology (Chongqing, China). The RNA interference (RNAi) sequences for pcDNA™6.2-GW/EmGFP-miR-β-catenin (5′-TGAACAAGACGTTGACTTGGA-3′) and the negative control pcDNA™6.2-GW/EmGFP-miR-β-catenin-n (5′-AAATGTACTGCGCGTGGAGAC-3′) were constructed by Invitrogen Life Technologies (Carlsbad, CA, USA). RPMI-l640 medium was purchased from HyClone Laboratories, Inc. (Logan, UT, USA). Fetal bovine serum (FBS) was purchased from Hangzhou Sijiqing Biological Engineering Materials Co., Ltd. (Hangzhou, China). Blasticidin was purchased from Sigma-Aldrich (St. Louis, MO, USA). Lipofectamine^®^ 2000 was purchased from Invitrogen Life Technologies. RNAiso Plus, reverse transcription-polymerase chain reaction (RT-PCR) amplification, and reverse transcription kits were all purchased from Takara Biotechnology Co., Ltd. (Dalian, China).

### Anti-HIF-1α (rabbit monoclonal antibody), anti-β-catenin (rabbit monoclonal antibody), anti-uPA (rabbit monoclonal antibody) and anti-MMP-7 (rabbit polyclonal antibody) were purchased from Epitomics (Burlingame, CA, USA)

Anti-GAPDH (rabbit monoclonal antibody), horseradish peroxidase (HRP)-conjugated goat anti-immunoglobulin G (IgG), radioimmunoprecipitation lysis buffer, phenylmethyl-sulfonyl fluoride, Bicinchoninic Acid (BCA) Protein Assay kit, Enhanced Chemiluminescence (ECL) reagent, goat anti-rabbit IgG and the immunohistochemistry streptavidin-peroxidase (SP) method chemical kit were purchased from Jiangsu BiYunTian Bio, Ltd. (Jiangsu, China).

### Cell culture and transfection

SGC-7901 human gastric cancer cells were cultured under normoxic conditions in RPMI-1640 medium supplemented with 10% fetal bovine serum (FBS) at 37°C in an atmosphere containing 5% CO2 and 25% O2. SGC-7901 cells were cultured under hypoxic conditions in RPMI-1640 medium supplemented with 10% fetal bovine serum (FBS) at 37°C, in the presence of 5% CO2, 1% O2 and 94% N2 for 16 h. For double hypoxic conditions, SGC-7901 cells were cultured with 150 *µ*mol/l CoCl2 (Sigma-Aldrich, St. Louis, MO, USA) (13) for 8 h concurrently with physical hypoxia for 16 h (2). All of the treatment and control groups were transfected with pcDNA™6.2-GW/EmGFP-miR-β-catenin or pcDNA™6.2-GW/EmGFP-miR-β-catenin-n in RPMI-1640 for 18 h at 37°C. A total of 1.6 *µ*g/ml blasticidin was used for selection, and the cells were screened after 6-8 weeks in order to obtain the stably transfected cell line miR-β-catenin-7901.

### Transwell invasion assays

The following five groups were included in the invasion assays: Control, liposome, negative control, hypoxia and hypoxia β-catenin knockdown. The cell migration and invasion assays of the cells were performed as described previously (14), using 8.0-*µ*m pore polycarbonate membrane Transwell inserts in a 24-well plate. The control group was cultured under normoxic conditions. The liposome group was cultured under normoxic conditions with the addition of 10 *µ*l liposome culture (Life Technologies, Carlsbad, CA, USA). The negative control group was cultured under normoxic conditions and transfected with the negative control plasmid pcDNA™6.2-GW/EmGFP-miR-β-catenin-n. The hypoxia group was cultured under physical hypoxic conditions. The hypoxia β-catenin knockdown group consisted of the stably transfected miR-β-catenin-7901 cell line cultured under physical hypoxic conditions.

To prepare the artificial basement membrane, Matrigel (40 *µ*l; Life Technologies) was evenly spread on a Boyden chamber membrane (Life Technologies), and placed into 12-well plates. SGC-7901 cells (2×10^5^) were seeded into the wells and cultured in RPMI-1640 supplemented with 5% FBS. The control, liposome and negative control groups were cultured for 24 h under normoxic conditions, whereas the hypoxic and hypoxic β-catenin knockdown groups were cultured for 8 h under normoxic conditions, and then cultured for 16 h under hypoxic conditions. The Boyden chamber was then removed, and the cells that remained on the upper surface were collected using a cotton swab. The cells remaining on the filter were fixed with 4% paraformaldehyde (Life Technologies) for 15 min, dried at room temperature, and stained with hematoxylin and eosin (Life Technologies). Images of the cells that had successfully migrated through the membrane were captured by microscopy (CKX41; Olympus, Tokyo, Japan) using a Canon camera (Canon, Japan). At least five different fields (magnification, ×200) were counted for each experiment, and the results of three independent experiments were averaged.

### RT-PCR detection of HIF-1α, β-catenin, uPA and MMP-7 mRNA expression levels

SGC-7901 cells were divided into the following groups: Control (48 h normoxia), hypoxia (32 h normoxia, physical hypoxia 16 h), and double hypoxia (24 h normoxia, concurrent 8 h chemical hypoxia and 16 h physical hypoxia). In addition, miR-β-catenin-7901 cells were divided into the following groups: Control β-catenin knockdown group, hypoxia β-catenin knockdown group and double hypoxia β-catenin knockdown group, all of which were cultured under the same conditions as listed above. Total RNA was extracted from the cells using RNAiso Plus according to the manufacturer's instructions. Reverse transcription to generate cDNA and PCR amplification were performed using a 7300 Real-Time PCR system (Applied Biosystems, Life Technologies, Thermo Fisher Scientific, Waltham, MA, USA). The cycle parameters were set as follows: 94°C for 5 min, then 94°C for 30 sec, 56°C (internal reference, β-actin), 55°C (HIF-1α), 57°C (β-catenin), 56°C (uPA) and 57°C (MMP-7) for 35 sec, and 72°C extension for 1 min for 30 cycles; and a final extension at 72°C for 5 min. The primer sequences are shown in Table I. PCR products were separated by 2.5% agarose gel electrophoresis and quantified using Quantity One 4.62 software (Bio-Rad Laboratories, Inc., Hercules, CA, USA) to analyze the gray value of the PCR products.

### Western blot analysis of HIF-1α, β-catenin, uPA and MMP-7 protein expression levels

Total protein was extracted from the six groups of cells, and the protein concentration was measured using the BCA method. Equal amounts of protein (50 *µ*g) were separated by SDS-PAGE (Life Technologies) and transferred to polyvinylidene fluoride membranes. The membranes were primarily blocked with 5% skimmed milk and then incubated with the primary antibodies targeting HIF-1α (1:5,000; rabbit monoclonal), β-catenin (1:10,000; rabbit monoclonal), uPA (1:3,000; rabbit monoclonal), MMP-7 (1:10,000; rabbit monoclonal) and GAPDH (1:1,000; rabbit monoclonal). All antibodies were purchased from Epitomics (Burlingame, CA, USA). The membranes were then incubated with HRP-conjugated secondary antibodies and visualized using the ECL reagent. The film was scanned using a gel imager (Bio-Rad Laboratories, Inc.) and Quantity One 4.62 software was used to analyze the gray values of the protein bands.

### Tumorigenicity assay and immunohistochemical staining in nude mice

BALB/c nude mice (n=5; 3 male, 2 female; 4–6 weeks old) were obtained from the Department of Laboratory Animal Science, Chongqing University Health Science Center (Chongqing, China). All animals were housed in standard conditions at 26–28°C, with 10 h light/14 h dull light and were given free access to food and water. All of the experiments were performed according to the animal protocol approved by the Institutional Animal Care and Use Committee of Chongqing Medical University (Chongqing, China). SGC-7901 cells were divided into the following groups: Control (48 h normoxia) and hypoxia (32 h normoxia, 16 h physical hypoxia). The miR-β-catenin-7901 cells were divided into a control β-catenin knockdown group and a hypoxia β-catenin knockdown group, which were cultured in the same manner as the SGC-7901 groups. The cells from each group were trypsinized, washed in phosphate-buffered saline (Life Technologies) and re-suspended in saline solution (Life Technologies). The nude mice were subcutaneously injected with 5×106 cells per 0.2 ml. Mice were divided into the control, hypoxia, interference and hypoxia interference groups, with five mice in each group. Tumor size was measured every third day. Tumor volume was calculated according to the following formula: V=(axb2)/2, where a was the largest superficial diameter and b the smallest superficial diameter. After four weeks, the mice were sacrificed by cervical dislocation, and the tumors were harvested and images were captured using a Canon IXUS 245 camera (Canon).

For the immunohistochemistry experiments, the tumors were harvested, fixed in formalin and embedded in paraffin (Life Technologies), and conventional immunohistochemistry sections were prepared. Detection was performed using the SP method in accordance with standard procedures (15). Positive expression was regarded as the presence of yellow-brown particles in cytoplasm or nucleus.

### Western blot analysis of protein expression levels in tumor tissues

Fresh tumor tissue from each group was harvested, and total protein was extracted using a protein extraction kit, according to the manufacturer's instructions. Western blots were performed as described above. Blots were quantified using grey scale analysis.

### Statistical analysis

Values are expressed as the mean ± standard error of the mean. The significance of differences between the groups was determined using Student's t-test and one-way analysis of variance. Appropriate post hoc tests were used when comparing multiple parameters. All analysis was performed using SPSS 17.0 software (IBM SPSS, Armonk, NY, USA). P<0.05 was considered to indicate a statistically significant difference.

## Results

### β-catenin knockdown decreases the invasiveness of gastric cancer cells under hypoxia

**Figure 1:**
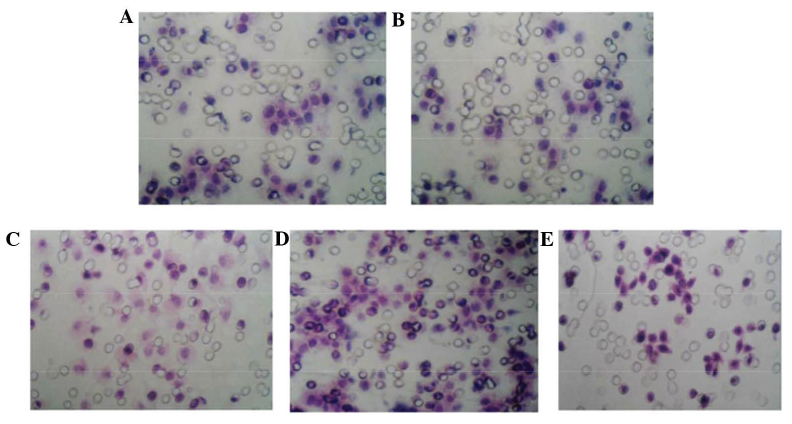
Invasive capacity of various groups of SGC-7901 gastric cancer cells (hematoxylin-eosin stained; magnification, ×200). (A) Control group; (B) liposome group; (C) negative control group; (D) hypoxia group; (E) hypoxia interference group. Representative photomicrographs (magnification, ×200) of cell invasion.

The invasion assay demonstrated that the number of invaded cells was not significantly different between the control, negative control and liposome groups. However, the number of invaded cells in the hypoxia group was increased as compared with that the control group, while the number in the hypoxia interference group was significantly decreased as compared with that in the hypoxia group (Table II and Fig. 1).

### Effects of β-catenin knockdown and hypoxia on HIF-1α, β-catenin, uPA and MMP-7 mRNA and protein expression levels

**Figure 2:**
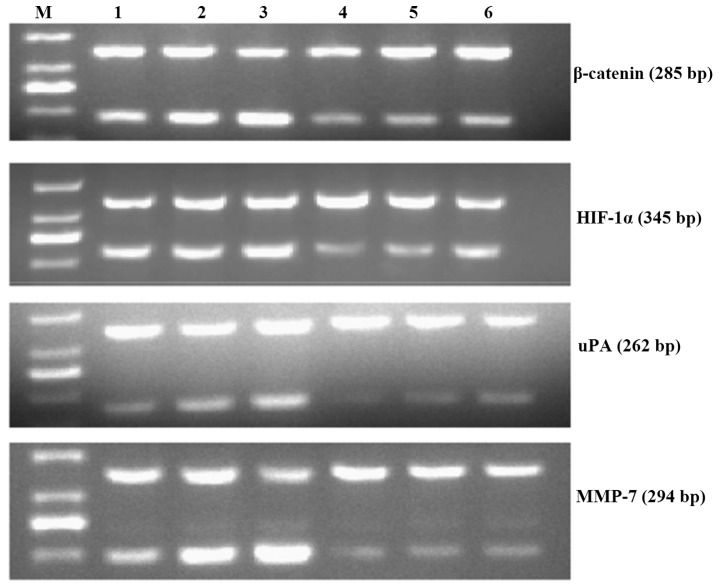
Representative images of HIF-1α, β-catenin, u-PA and MMP-7 mRNA expression in each of the SGC-7901 gastric cancer cell groups. M, DNA marker DL1,000; 1, control group; 2, hypoxia group; 3, double hypoxia group; 4, interference group; 5, hypoxia interference group; 6, double hypoxia interference group. HIF-1α, hypoxia-inducible factor-1α; uPA, urokinase-type plasminogen activator; MMP-7, matrix metalloproteinase-7.

**Figure 3:**
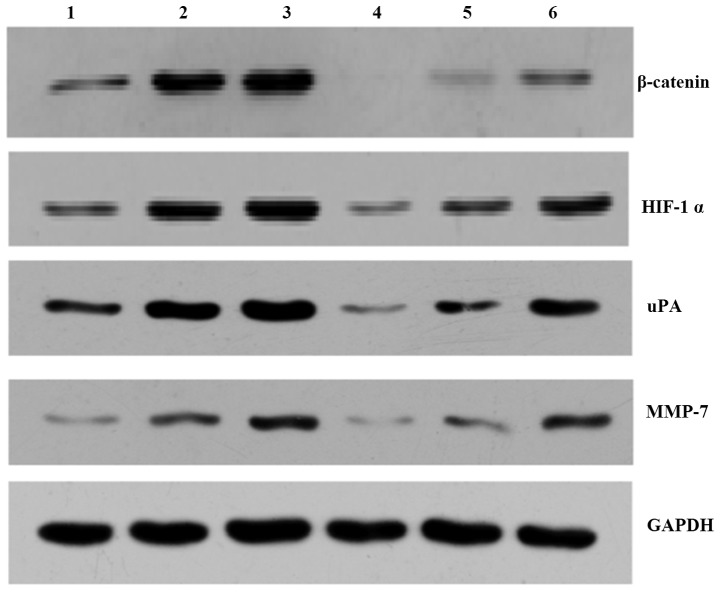
Representative images of HIF-1α, β-catenin, u-PA and MMP-7 protein expression in each of the SGC-7901 gastric cancer cell groups. 1, control group; 2, hypoxia group; 3, double hypoxia group; 4, interference group; 5, hypoxia interference group; 6, double hypoxia interference group. HIF-1α, hypoxia-inducible factor-1α; uPA, urokinase-type plasminogen activator; MMP-7, matrix metalloproteinase-7.

RT-PCR and western blot analyses demonstrated that the mRNA and protein expression levels of β-catenin, HIF-1α, uPA and MMP-7 were low in the SGC-7901 control group, while they were significantly enhanced in the hypoxia group, and in the double hypoxia to an even greater extent (P<0.05) (Tables III and IV, Figs. 2 and 3). The control β-catenin knockdown (interference) group expressed significantly lower levels of β-catenin, HIF-1α, uPA and MMP-7 mRNA and protein as compared with those in the control group. In addition, the hypoxia β-catenin knockdown group expressed significantly lower levels of β-catenin, HIF-1α, uPA and MMP-7 mRNA and protein as compared with those in the hypoxia and control groups (P<0.05). The hypoxia β-catenin knockdown group expressed significantly lower levels of β-catenin, HIF-1α, uPA and MMP-7 mRNA and protein as compared with those in the double hypoxia interference group.

The mRNA and protein expression levels of β-catenin, HIF-1a, uPA and MMP-7 were increased in the hypoxia β-catenin knockdown group and the double hypoxia β-catenin knockdown group, as compared with those in the control β-catenin knockdown group (P<0.05) (Tables III and IV, Figs. 2 and 3).

Compared to the double hypoxia β-catenin knockdown group to the hypoxia β-catenin knockdown group, uPA and MMP-7 mRNA and protein expression levels were significantly increased (P<0.05) (Table V).

### Hypoxia accelerates growth of gastric xenograft tumors

**Figure 4:**
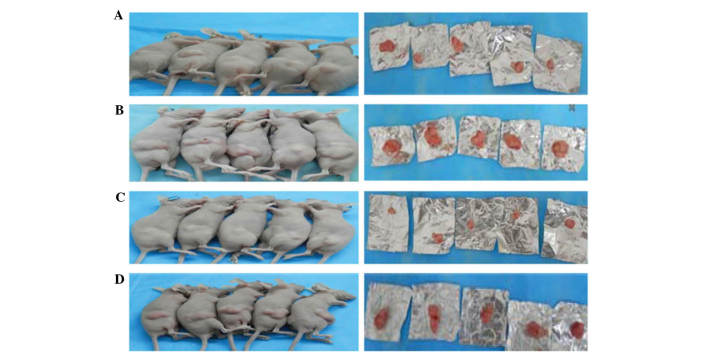
Suppression of tumor growth by interfering β-catenin *in vivo*. The average volume and weight of the xenografts in the hypoxia group were significantly lower as compared with those in the hypoxia interference group (P<0.05), which is concordant with the *in vitro* findings. (A) Control group; (B) hypoxia group; (C) interference group; (D) hypoxia interference group.

Ten days following inoculation of the cells into nude mice, the cells from the hypoxia group began to form tumors. After 14 days, the cells from the control group also began to form tumors. Finally, after 16 days, the control and hypoxia β-catenin knockdown groups began to form tumors. Following inoculation, time-dependent increases in tumor volume were observed, which were accelerated after 22 days. According to the tumor volume and speed of growth, the tumorigenicity of the hypoxia group was higher as compared with that in the control group and the β-catenin knockdown group (Table VI, Fig. 4).

### Immunohistochemical analysis of HIF-1α, β-catenin, uPA and MMP-7 expression in nude mouse xenografts

**Figure 5:**
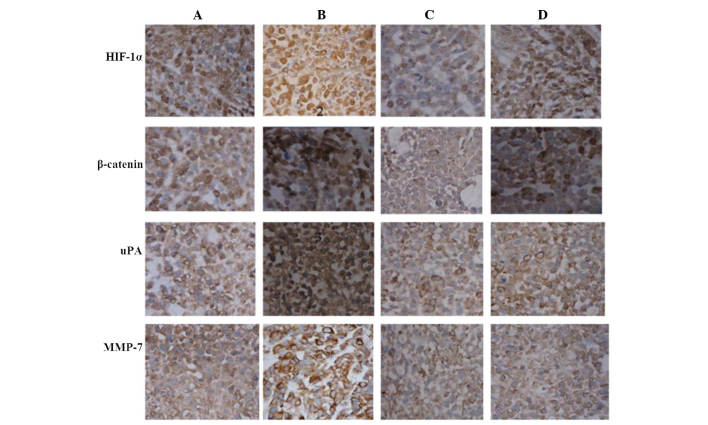
Expression of HIF-1α, β-catenin, u-PA and MMP-7 in xenograft tumors, as determined by immunohistochemistry. (A) Control group; (B) hypoxia group; (C) interference group; (D) hypoxia interference group (magnification, ×400). HIF-1α, hypoxia-inducible factor-1α; uPA, urokinase-type plasminogen activator; MMP-7, matrix metalloproteinase-7.

Four weeks after inoculation, the nude mice were sacrificed. The tumors were embedded in paraffin and immunohistochemistry detected HIF-1α, β-catenin and uPA expression predominantly in the nucleus, while MMP-7 expression was primarily located in the nucleus and the cytoplasm (Fig. 5). A lower protein expression levels was observed in the interference group compared with the control group.

### Western blot analysis of protein expression levels in tumor tissue

**Figure 6:**
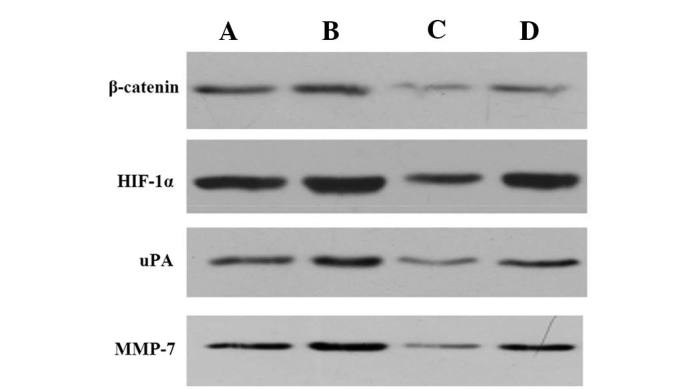
Representative Western blot of HIF-1α, β-catenin, u-PA and MMP-7 protein levels in xenograft tumors. (A) Control; (B) hypoxia; (C) interference; and (D) hypoxia interference groups. HIF-1α, hypoxia-inducible factor-1α; uPA, urokinase-type plasminogen activator; MMP, matrix metalloproteinase.

HIF-1α, β-catenin, uPA and MMP-7 protein expression levels were highest in the tumor tissue from the hypoxia group and lowest in the interference group. Expression levels of these proteins were significantly reduced in the hypoxia interference group, as compared with those in the hypoxia group (P<0.05) (Fig. 6, Table VII).

## Discussion

The carcinogenic activity of the Wnt/β-catenin pathway relies on the accumulation of cytosolic β-catenin. The Wnt signaling pathway is regulated by the levels of β-catenin in the cell; when the levels of β-catenin are elevated, the Wnt pathway is activated (16). HIF-1α stabilization depends on the phosphoinositide 3-kinase (PI3K)/Akt pathways, which are involved in the transcriptional activity of the extracellular signal-regulated kinase (ERK) pathway (17). The Wnt/β-catenin pathway communicates with the PI3K/Akt pathway (18), and β-catenin is also involved in activation of the ERK pathway (19). These observations suggested that increased HIF-1 expression and activity may be associated with the Wnt/β-catenin signaling pathway.

Following suppression of β-catenin expression using RNAi, β-catenin and HIF-1α expression levels were significantly reduced, indicating that HIF-1α levels dropped due to the decrease in β-catenin. These results are concordant with the findings of Kaidi *et al* (20) and Lee *et al* (21). The results of the present study suggested that HIF-1α may function downstream of Wnt/β-catenin and may be regulated by the Wnt/β-catenin signaling pathway. Conversely, in the double hypoxia and hypoxia groups, HIF-1α and β-catenin expression levels were increased as compared with those in the control group. Furthermore, following suppression of β-catenin expression, the levels of β-catenin did not remain constant in the stably transfected cell line. The hypoxia β-catenin knockdown group and the double hypoxia β-catenin knockdown group had increased expression levels of β-catenin, as compared with those in the control β-catenin knockdown group. It may therefore be hypothesized that hypoxia can stimulate an increase in HIF-1α, and that HIF-1α can activate β-catenin, stimulating the Wnt/β-catenin signaling pathway and activating downstream genes. Jiang *et al* (22) previously demonstrated, by western blot and RT-PCR analyses, that in prostate cancer cells, HIF-1α regulated the expression levels of β-catenin. Furthermore, Lim *et al* (23) suggested that HIF-1α may interact with hARD1 to regulate β-catenin.

A previous study by our group indicated that hypoxia is able to increase HIF-1α expression, which upregulates MMP-9 and uPA receptor expression in gastric cancer cells, resulting in increased adhesion, migration and invasion (2). In the present study, western blot and RT-PCR analyses demonstrated that in the hypoxia and double hypoxia groups, uPA and MMP-7 mRNA and protein expression levels gradually increased. Following knockdown of β-catenin expression, the hypoxia β-catenin knockdown group, as compared with the hypoxia group, as well as the double hypoxia β-catenin knockdown group, as compared with the double hypoxia group, had significantly decreased expression levels of uPA and MMP-7 mRNA and protein. Under hypoxic conditions and following treatment with cobalt chloride in order to increase the effectiveness of hypoxia, followed by β-catenin knockdown, the double hypoxia β-catenin knockdown group and the hypoxia β-catenin knockdown group had significantly elevated expression levels of uPA and MMP-7, as compared with those in the hypoxia β-catenin knockdown group and the β-catenin knockdown group. This suggested that suppression of β-catenin expression influenced MMP-7 and uPA expression levels, and under hypoxic conditions, HIF-1α was capable of regulating the expression of uPA and MMP-7, based on the magnitude of the enhanced hypoxia.

According to the in vitro invasion assay, the number of cells transgressing the membrane was increased in the hypoxia group, and invasion of the gastric cancer cells under hypoxic conditions was enhanced. Following knockdown of β-catenin expression, invasiveness was reduced under hypoxia. These results indicated that the absence of oxygen in the environment promotes the expression of HIF-1α, which increases β-catenin expression and activate the Wnt/β-catenin signaling pathway in gastric cancer cells, resulting in increased invasiveness.

In the nude mouse xenograft experiment, tumors from the hypoxia group were significantly larger as compared with those from the control group and the hypoxia β-catenin knockdown group, indicating that tumor growth was enhanced for the hypoxia group, as compared with that in the control group. The immunohistochemistry and western blotting results demonstrated that in the xenograft tumors from the hypoxia group, HIF-1α, β-catenin, uPA and MMP-7 protein expression levels were higher as compared with those in the control group and the hypoxia β-catenin knockdown group.

In conclusion, the in vitro transwell invasion assay capacity of gastric cancer cells is enhanced under hypoxic conditions when HIF-1α expression is increased by activating the Wnt/β-catenin signaling pathway, subsequently inducing the expression of uPA and MMP-7, which act on the extracellular matrix, promoting cell invasion and the development of gastric cancer.

In the present study, RT-PCR and western blot analyses demonstrated that a regulatory interaction exists between the Wnt/β-catenin pathway and HIF-1α. Concurrently, inhibiting β-catenin expression under hypoxic conditions reduced the size of the xenograft tumor as well as uPA and MMP-7 protein expression levels. These results suggested that the ability to enhance gastric cancer cell invasion under hypoxic conditions may be due to an increase in HIF-1α expression, which is real-ized through the activation of Wnt/β-catenin signaling, which can promote uPA and MMP-7 expression. uPA and MMP-7 may then degrade the extracellular matrix in order to promote tumor invasion, thereby contributing to gastric cancer invasiveness.

The present study suggested that in gastric cancer cells, HIF-1α has a role both upstream and downstream of the Wnt/β-catenin signaling pathway, and that these pathways have a reciprocal regulative function. Under hypoxic conditions, HIF-1α can facilitate the increased expression of uPA (24) and MMP-7 (25–27) through the Wnt/β-catenin pathway, which can promote the invasiveness of gastric cancer cells. These results may provide a basis for gastric cancer treatment at the molecular level.

## Figures and Tables

**Table I:** Primers used for polymerase chain reaction amplification.

Gene	Primer sequence	Product size (bp)
β-catenin	R: 5-GCCATTACAACTCTCCACAACC-3 F: 5-GACAGATAGCACCTTCAGCACTC-3	285
HIF-1α	R: 5-CCAACAGTAACCAACCTCAGTG-3 F: 5-CAACCCAGACATATCCACCTCT-3	345
uPA	R: 5-GCTTGCTCACCACAACGACA-3 F: 5-CCTTGGAGGGAACAGACGAG-3	262
MMP-7	R: 5-GAAGCCAAACTCAAGGAGATGC-3 F: 5-GTCAGCAGTTCCCCATACAACT-3	294
β-actin	R: 5-GACCCAGATCATGTTTGAGACC-3 F: 5-ATCTCCTTCTGCATCCTGTCG-3	594

HIF-1α, hypoxia-inducible factor-1α; uPA, urokinase-type plasminogen activator; MMP-7, matrix metalloproteinase-7; R, reverse; F, forward; bp, base pair.

**Table II:** Invasive capacity of various groups of SGC-7901 gastric cancer cells.

Group	Invaded cells (n)
Control	66±3
Liposome	62±5
Negative control	65±3
Hypoxia	118±4^a^
Hypoxic interference	50±2^b^

Values are expressed as the mean ± standard error of the mean (n=3).

a P<0.05, as compared with the control group;

b P<0.05, as compared with the hypoxia group.

**Table III:** mRNA expression levels of HIF-1α, β-catenin, uPA and MMP-7 in each of the SGC-7901 gastric cancer cell groups.

Group	β-catenin	HIF-1α	uPA	MMP-7
Control	0.49±0.03	0.51±0.05	0.50±0.03	0.75±0.04
Hypoxia	0.76±0.06^a^	0.74±0.07^a^	0.69±0.05^a^	1.15±0.14^a^
Double hypoxia	1.25±0.09^b^,^a^	0.99±0.08^b^,^a^	0.80±0.07^b^,^a^	1.70±0.04^b^,^a^
Interference	0.23±0.04^a^	0.25±0.04^a^	0.36±0.03^a^	0.38±0.08^a^
Hypoxia interference	0.31±0.02^b^,^d^	0.41±0.03^b^,^d^	0.45±0.04^b^,^d^	0.56±0.05^b^,^d^
Double hypoxia interference	0.42±0.05^b^,^c^,^d^	0.78±0.59^b^,^c^,^d^	0.54±0.08^b^,^c^,^d^	0.64±0.04^b^,^c^,^d^

Values are expressed as the mean ± standard error of the mean (n=3).

a P<0.05, as compared with the control group;

b P<0.05, as compared with the hypoxia group;

c P<0.05, as compared with the double hypoxia group;

d P<0.05, as compared with the interference group. HIF-1α, hypoxia-inducible factor-1α; uPA, urokinase-type plasminogen activator; MMP-7, matrix metalloproteinase-7.

**Table IV:** Protein expression levels of HIF-1α, β-catenin, uPA and MMP-7 protein in the SGC-7901 gastric cancer cell groups.

Group	β-catenin	HIF-1α	uPA	MMP-7
Control	0.60±0.06	0.50±0.06	0.27±0.12	0.64±0.14
Hypoxia	1.09±0.18^a^	0.90±0.22^a^	0.51±0.05^a^	1.49±0.20^a^
Double hypoxia	1.89±0.21	1.59±0.36	0.88±0.11^b^,^a^	1.98±0.49^b^,^a^
Interference	0.11±0.03	0.10±0.01	0.07±0.05^a^	0.15±0.04^a^
Hypoxia interference	0.33±0.05	0.48±0.13	0.33±0.05^b^,^d^	0.58±0.04^b^,^d^
Double hypoxia interference	0.59±0.01	1.00±0.25	0.56±0.08^b^,^c^,^d^	1.02±0.11^b^,^c^,^d^

Values are expressed as the mean ± standard error of the mean (n=3).

a P<0.05, as compared with the control group;

b P<0.05, as compared with the hypoxia group;

c P<0.05, as compared with the double hypoxia group;

d P<0.05, as compared with the interference group. HIF-1α, hypoxia-inducible factor-1α; uPA, urokinase-type plasminogen activator; MMP-7, matrix metalloproteinase-7.

**Table V:** mRNA expression levels of uPA and MMP-7 in each of the SGC-7901 gastric cancer cell groups.

Group	uPA	MMP-7
Hypoxia interference group-interference group	0.09±0.03	0.10±0.08
Double hypoxia interference group-hypoxia interference group	0.15±0.04^a^	0.18±0.06^a^

Values are expressed as the mean ± standard error of the mean (n=3).

a P<0.05, as compared with the hypoxia interference group-interference group. uPA, urokinase-type plasminogen activator; MMP-7, matrix metalloproteinase-7.

**Table VI:** Average volume and weight of xenograft tumors in four groups.

Group	Volume (m^3^)	Weight (g)
Control	1232±56	0.61±0.03
Hypoxia	1273±48^a^	1.20±0.07^a^
Interference	334±16^a^	0.37±0.05^a^
Hypoxia interference	683±39^b^,^c^	0.82±0.03^b^,^c^

Values are expressed as the mean ± standard error of the mean (n=5).

a P<0.05, as compared with the control group;

b P<0.05, as compared with the hypoxia group;

c P<0.05, as compared with the interference group.

**Table VII:** Protein levels of HIF-1α, β-catenin, uPa and MMP-7 in nude mouse xenografts.

Group	β-catenin	HIF-1α	uPA	MMP-7
Control	1.61±0.03	2.82±0.09	1.86±0.05	1.94±0.03
Hypoxia	2.06±0.09^a^	3.68±0.09^a^	2.92±0.06^a^	2.87±0.07^a^
Interference	0.57±0.06^a^	1.86±0.11^a^	1.12±0.06^a^	0.81±0.07^a^
Hypoxia interference	1.10±0.08^b^,^c^	2.07±0.15^c^	1.97±0.04^b^,^c^	1.8±0.06^b^,^c^

Values are expressed as the mean ± standard error of the mean (n=3).

a P<0.05, as compared with the control group;

b P<0.05, as compared with the hypoxia group;

c P<0.05, as compared with the interference group. HIF-1α, hypoxia-inducible factor-1α; uPA, urokinase-type plasminogen activator; MMP-7, matrix metalloproteinase-7.
